# Epistemic Barriers to Buprenorphine Access for Adolescents With Opioid Use Disorder

**DOI:** 10.7759/cureus.108824

**Published:** 2026-05-14

**Authors:** Arya Babul, Sohi Ashraf, Russell Elmes, Parisa Mahdavi

**Affiliations:** 1 Biomedical Sciences, West Career and Technical Academy, Las Vegas, USA; 2 Biomedical Sciences, Society for Awareness of Neglected Diseases, Las Vegas, USA; 3 Critical Care Medicine, MountainView Hospital, Las Vegas, USA; 4 Emergency Medicine, West Henderson Hospital, Henderson, USA; 5 Drug Development, Tavolar LLC, Las Vegas, USA

**Keywords:** adherence, adolescents, buprenorphine, epistemic injustice, extended release, health disparities, medication for opioid use disorder, opioid use disorder, treatment access, youth substance use treatment

## Abstract

Adolescents with opioid use disorder (OUD) remain vulnerable to fentanyl exposure, opioid‑related harms, and profound treatment gaps, even with recent declines in overdose mortality. Strong evidence demonstrates that buprenorphine is safe and effective for youth, yet adolescents remain the age group least likely to receive medications for OUD (MOUD). This review reassesses buprenorphine access for adolescents through a risk-benefit lens, integrating randomized trials, observational cohorts, international guidelines, and emerging fentanyl‑era data.

We synthesized evidence from randomized controlled trials, administrative datasets, qualitative studies, comparative effectiveness research, and narrative reviews through a search of the English-language literature in PubMed for adolescent and young adults; examined U.S., U.K., and WHO policy positions; and evaluated structural, regulatory, and developmental barriers that limit adolescent access to MOUD. Particular attention was given to transmucosal and extended‑release buprenorphine and its potential relevance to adolescent adherence and retention.

Across two decades of research, buprenorphine consistently improves retention, reduces opioid use, decreases high‑risk behaviors, and is well tolerated. Longer treatment durations reliably outperform short tapers. Real‑world data show that only 5 to 10% of adolescents with OUD receive MOUD, despite the highest overdose risk of any age group. Comparative effectiveness studies demonstrate lower overdose risk with buprenorphine than methadone, and fentanyl‑era feasibility studies confirm successful induction even among youth using illicit fentanyl exclusively. All major medical organizations, including American Society of Addiction Medicine, the American Academy of Pediatrics, the American Academy of Child & Adolescent Psychiatry, the Society for Adolescent Health and Medicine, the British Columbia Centre on Substance Use, and the WHO, endorse buprenorphine for adolescents with OUD. Persistent undertreatment reflects structural barriers, clinician hesitancy, regulatory misconceptions, and the absence of an FDA indication, not lack of efficacy.

Expanding access to both transmucosal and extended‑release formulations is critical to reducing preventable overdose deaths. The evidence supports a shift toward developmentally tailored, medication‑based recovery models that prioritize early initiation, sustained treatment, and removal of structural barriers. With illicitly manufactured synthetic opioids now dominating the drug supply, withholding buprenorphine from adolescents is not evidence‑based; it reflects longstanding epistemic exclusion that has limited youth access to treatments known to save lives.

## Introduction and background

“When in doubt, do not taper.” 

- Chang DC et al., Medication-Assisted Treatment for Youth with Opioid Use Disorder, Am J Drug Alcohol Abuse, 2018

This narrative review examines the evidence for buprenorphine treatment in adolescents with opioid use disorder (OUD) and the multilevel factors that shape access.

The persistent undertreatment of adolescents with OUD

Despite clear and accumulating evidence that buprenorphine is safe and effective for adolescents with OUD, structural, regulatory, and cultural barriers continue to deny adolescents access to the only medication with randomized evidence. This persistent pattern of undertreatment carries serious and preventable consequences, including overdose, death, and the entrenchment of chronic opioid addiction into adulthood. Throughout this review, we use terms such as epistemic barriers, epistemic injustice, and epistemic exclusion in a broad analytic sense that encompasses not only knowledge‑ and perception‑related factors but also the structural, regulatory, developmental, and cultural forces that shape whose evidence is recognized, whose risks are prioritized, and whose treatment needs are systematically overlooked.

Drawing on expert consensus statements and clinical guidelines from the American Society of Addiction Medicine (ASAM) [1], the American Academy of Pediatrics (AAP) [2], the American Academy of Child & Adolescent Psychiatry (AACAP) [3], the British Columbia Centre on Substance Use (BCCSU) Youth Guideline [4,5], the Society for Adolescent Health and Medicine (SAHM) [6], and the Brown Evidence‑Based Practice Center (EPC)/Agency for Healthcare Research and Quality (AHRQ) [7], as well as recent contributions from Kleinman [8], Woody [9], Marsch [10,11], Neptune and Kaliamurthy [12], Hadland [13-17], and Welsh [18-21], this review examines the evidence supporting buprenorphine as a core element of care for adolescents (ages 12-17) with moderate to severe OUD.

It also examines the persistent barriers that limit adolescent access to buprenorphine, the consequences of these barriers, the emerging relevance of medication‑based recovery (MBR) [22,23], the critical research gaps that remain, and the potential promise and limitations of the proposed NIH HEAL Initiative’s ‘Extended‑Release Buprenorphine for Adolescents (ERA)’ trial (NIDA CTN‑0158), led by Fishman, Marsch, and Nunes [24].

Regulatory definitions of adolescence

In U.S. regulatory contexts, the FDA defines adolescents as individuals 12 to 17 years of age, consistent with the pediatric age groupings outlined in the FDA’s Clinical Pharmacology Considerations for Pediatric Studies guidance [25], Pediatric Study Plans guidance [26], the ICH E11(R1) pediatric framework adopted by the FDA [27], and FDA’s Pediatric Drug Development: Regulatory Considerations [28]. This age range is also widely used in FDA‑approved prescribing information to define adolescent indications, stratify safety and efficacy data, and structure pediatric labeling. Despite this explicit regulatory demarcation, research frequently collapses these categories by grouping adolescents with young adults (18-22, 18-24, or 18-25) under broad labels such as “youth". While this practice may boost sample size and is often justified by perceived pharmacokinetic and pharmacodynamic similarity, it erodes the regulatory distinction that governs pediatric labeling and limits the ability to generate the age‑specific evidence needed to support an adolescent indication.

Medication‑based recovery as a developmental framework

MBR, recently championed by McLellan and Volkow [22], offers a clinically realistic and ethically grounded framework that is arguably even more essential for adolescents than for adults. Adolescents with OUD face the highest overdose mortality in history, yet remain the least likely to receive evidence‑based medications, the least likely to be retained in care, and the most likely to be discharged for continued use, a pattern that represents epistemic indifference at best and epistemic injustice at its worst.

MBR rejects abstinence‑contingent treatment models that punish relapse, a developmental inevitability in youth, and instead emphasizes protection, remission, and recovery while patients remain on medication [22,23]. This staged approach aligns with the adolescent evidence base. Applying MBR to adolescents reframes urine positive for non‑prescribed opioids not as a failure warranting taper or discharge, but as a clinical signal requiring intensified support, just as persistent hyperglycemia prompts treatment adjustment rather than treatment cessation. In this sense, MBR is not merely appropriate for adolescents; it is a clinical and moral imperative. By prioritizing safety, continuity, and developmentally attuned care, MBR provides a unifying framework that supports the expansion of buprenorphine access, including extended‑release formulations, and counters the epistemic exclusion that has long marginalized youth treatments that save adult lives.

Historical and regulatory drivers of evidence gaps

The limited evidence base for buprenorphine use in adolescents with OUD, particularly for extended‑release formulations, reflects historical, regulatory, and cultural factors rather than any inherent clinical unsuitability of the medication. This pattern is not unique to OUD; across many therapeutic areas, pediatric and adolescent data remain sparse due to ethical constraints on research involving minors, practical challenges in recruiting adequately powered samples, and longstanding commercial disincentives to conduct pediatric trials. Much of the gap in buprenorphine research stems from the timing of product availability. Transmucosal buprenorphine for OUD, recently described by the FDA as “buprenorphine‑containing transmucosal products for the treatment of opioid dependence” (BTODs), entered the market in 2002, roughly 15 years before the first extended‑release monthly injectable from Indivior (Sublocade, NDA 209819) [29] and nearly two decades before the weekly and monthly formulation from Braeburn (Brixadi, NDA 210136) [30]. As a result, the evidence base accumulated first and most extensively for the older transmucosal formulations, while extended‑release products entered a research landscape shaped by entrenched assumptions and outdated regulatory language.

Entrenched misstatements and their downstream effects

For many years, the prescribing information for transmucosal buprenorphine stated that “safety and effectiveness … in patients below the age of 16 have not been established.” Although this language was quietly changed around 2018 to “safety and effectiveness … has not been established in pediatric patients,” apparently without any publicly available regulatory correspondence or clarification, its legacy persists. More than two dozen guidelines and peer‑reviewed publications continue to assert, incorrectly, that transmucosal buprenorphine is “approved by the FDA” only for patients aged 16 and older. These misstatements appear in influential documents, including AACAP’s guideline [3], ASAM’s National Practice Guideline [1], AAP publications [2], the JAACAP Revised Principles and Practice Recommendations [20], and a recent state‑of‑the‑art review in JAMA Pediatrics [31]. Over time, this misinformation has become embedded in secondary sources, shaping clinician behavior long after the underlying regulatory language changed.

Importantly, no buprenorphine formulation is FDA‑approved for the treatment of OUD in individuals under 18, a position that contrasts with regulatory frameworks elsewhere. In the European Union and United Kingdom, transmucosal buprenorphine is indicated for OUD in adults and adolescents aged 15 years and older, and extended‑release buprenorphine is indicated for adults and adolescents aged 16 years and older. These international indications underscore that the U.S. evidence gap reflects historical and regulatory inertia rather than any inherent clinical unsuitability of buprenorphine for adolescents.

Cultural hesitancy and treatment disparities

Other factors reinforced the dominance of transmucosal buprenorphine in the literature. Clinicians have historically viewed transmucosal administration as more acceptable for adolescents, although widespread adoption of subcutaneous therapies across other chronic diseases has shifted perceptions of injection‑based treatments. Transmucosal buprenorphine can also be initiated with limited supervision and does not require a test dose except in specific scenarios, whereas extended‑release buprenorphine requires a test dose and a more complex initiation pathway.

As demonstrated by Schuler et al. [32], buprenorphine treatment increased more than threefold among adults over 55 but declined among youth under 18, even as overdose deaths surged. National Treatment Episode Data Set (TEDS) data show that medications for OUD (MOUD) are planned at admission for 93% of adults aged 25 and older, 56% of young adults aged 18-24, and only 2% of adolescents aged 12-17 [18]. More recent TEDS‑Admissions (TEDS-A) data confirm significantly lower odds of planned MOUD use among adolescents [33]. Cano et al. reported that only 30.8% of adolescents with OUD received any past‑year substance use treatment, and MOUD was included in just 9.5% of adolescent admissions compared with 36.4% of adult admissions [34]. Adolescents continue to be directed toward psychosocial interventions and short‑term detoxification, approaches repeatedly shown to be insufficient, particularly in the fentanyl era [3,4,13,14,35,36]. Surveys of addiction treatment staff further illustrate this gradient: enthusiasm for MOUD declines sharply for adolescents aged 16-17 [18,19,37,38].

Consequences of the adolescent treatment gap

The lack of an indication remains the ‘elephant in the room,’ shaping family reticence as well as clinician behavior, guideline development, and research priorities. It also contributes to payer‑driven barriers, including coverage restrictions, reimbursement limitations, and prior‑authorization delays, and reinforces clinician concerns about potential regulatory scrutiny. It is not evidence that has constrained adolescent access to buprenorphine, but rather the inertia of outdated regulatory language, persistent misconceptions, and longstanding discomfort with treating youth using the same evidence‑based medications routinely offered to adults.

The objective of this review is to synthesize current knowledge on buprenorphine treatment for adolescents with OUD and the developmental factors that shape access.

## Review

Methods

This review was based on PubMed searches for peer‑reviewed, English‑language articles related to pediatric OUD, overdose reversal, and MOUD. Search terms included opioid‑related disorders combined with keywords such as pediatric, adolescent, youth, overdose, mortality, complications, diagnosis, drug therapy, methadone, buprenorphine, fentanyl, naloxone, epidemiology, etiology, pathophysiology, rehabilitation, therapy, clinical practice guidelines, and federal regulation. Searches were conducted through April 7, 2026. To identify additional eligible references, we manually reviewed the bibliographies of identified articles. No date limits were applied. Given the scarcity of prospective randomized trials in adolescents, a systematic review was not feasible; therefore, this work was conducted as a narrative review.

Agency positions on MOUD for adolescents: U.S. and international perspectives

U.S. Federal Positions

Federal public health agencies have articulated a clear policy stance: adolescents with OUD should have access to buprenorphine‑based treatment, and age alone must not restrict evidence‑based care. Although these positions have not yet translated into routine clinical practice, they establish the scientific, ethical, and regulatory foundation for understanding the current evidence landscape and highlight the persistent gap between policy intent and real‑world treatment patterns.

In the U.S., the National Institute on Drug Abuse (NIDA) offers one of the strongest federal endorsements of buprenorphine treatment for youth. Its clinical resource, "Medication Treatment for Opioid Use Disorder in the Pediatric (Adolescent Medicine) Setting", explicitly encourages pediatricians to diagnose and treat adolescent OUD rather than defer to adult‑oriented addiction programs [39]. NIDA emphasizes that buprenorphine improves retention and reduces opioid use and should be delivered within a developmentally informed framework that incorporates psychosocial supports, structured scheduling, and family engagement.

Central to NIDA’s position is the pivotal NIDA Clinical Trials Network study by Woody et al. [9], the most rigorous randomized evidence available for buprenorphine treatment in adolescents and young adults with OUD. In this multicenter study of 152 participants aged 15-21, a 12‑week buprenorphine-naloxone maintenance regimen outperformed a 14‑day taper. Youth receiving buprenorphine maintenance demonstrated far fewer nonprescribed opioid-positive urine tests, higher retention (70% vs 20.5% at week 12), and lower rates of injecting and other high‑risk behaviors. Buprenorphine was safe and well tolerated, with no major adverse events. The detoxification group, by contrast, experienced rapid relapse and early disengagement. Together, NIDA’s guidance and the Woody trial establish a clear empirical conclusion: short‑term detoxification is clinically inadequate for adolescents, whereas continued buprenorphine treatment improves outcomes across every meaningful domain.

The Substance Abuse and Mental Health Services Administration (SAMHSA) echoes this position [40]. In its national guidance on MOUD, SAMHSA explicitly supports buprenorphine as an evidence‑based treatment option for adolescents and directly cites the Woody trial as foundational evidence. The alignment between the NIDA and SAMHSA reflects a federal consensus: age should not be used as a rationale to withhold buprenorphine, particularly given the escalating overdose risk facing adolescents.

World Health Organization Guidance

International guidance reinforces this stance. The WHO’s Guidelines for the Psychosocially Assisted Pharmacological Treatment of Opioid Dependence explicitly state that opioid agonist therapy, including buprenorphine, is effective for OUD and should be accessible to adolescents [41]. The WHO emphasizes that age alone should not preclude evidence‑based pharmacologic treatment and that adolescents with OUD face substantial morbidity and mortality risks. Accordingly, the WHO recommends that youth “should have access to the same evidence‑based treatments available to adults,” delivered with appropriate developmental considerations.

WHO’s overdose prevention guidance identifies opioid agonist therapy as a core component of reducing fatal overdose risk, and its consolidated HIV prevention guidelines recommend offering opioid substitution therapy, including buprenorphine, to adolescents who inject opioids [42].

U.K. Positions

Compared with the U.S., formal guidance from U.K. bodies on pharmacologic treatment of OUD in adolescents is less detailed but directionally aligned. Across agencies and professional societies, three themes recur: buprenorphine and methadone are appropriate for older adolescents, maintenance therapy is preferred over detoxification, and age alone should not deny access to opioid agonist therapy.

In the U.K., the National Institute for Health and Care Excellence (NICE) explicitly includes 16‑ and 17‑year‑olds within its evidence‑based treatment framework for OUD [43]. NICE recommends methadone or buprenorphine as first‑line pharmacologic options for individuals aged 16 and older, with the choice driven by clinical factors rather than age. Maintenance therapy is generally preferable to detoxification for those at high risk of relapse, a category that encompasses nearly all adolescents with OUD. Consistent with this clinical guidance, both the European Medicines Agency (EMA) and the U.K. Medicines and Healthcare products Regulatory Agency (MHRA) authorize transmucosal buprenorphine for OUD in adults and adolescents aged 15 years and older, and extended‑release buprenorphine for adults and adolescents aged 16 years and older.

The U.K. Advisory Council on the Misuse of Drugs (ACMD) reinforces this stance, emphasizing that young people should have access to the full range of pharmacologic treatments, including buprenorphine, and warning that withholding opioid agonist therapy increases overdose risk, particularly in fentanyl‑adulterated drug markets [44].

A Convergent International Consensus

Taken together, U.S., WHO, and U.K. positions form a coherent and authoritative framework: buprenorphine is essential for adolescents with OUD. These conclusions emerge from health systems with different regulatory cultures yet converge on the same core principle: adolescents should have access to buprenorphine‑based maintenance treatment, and the current treatment gap reflects system‑level barriers rather than a lack of evidence. Although scientific evidence, federal policy, and global public health consensus are aligned, clinical practice has not kept pace. This disconnect between guidance and implementation remains one of the central challenges in adolescent OUD treatment and underscores the urgency of aligning real‑world care with the standards articulated by national and international health agencies.

Professional society guidelines

Consensus Across Professional Societies

Despite persistent misconceptions, outdated regulatory language, and uneven implementation across clinical settings, professional guidance is unequivocal that age alone must not be used as a barrier to initiating pharmacotherapy in youth. Yet in practice, adolescents continue to face substantial obstacles to receiving these treatments. High‑risk youth rarely receive prescriptions for naloxone, and dispensing rates for both naloxone and buprenorphine remain far below those of adults, reflecting structural barriers that blunt the impact of even the strongest professional recommendations [45,46].

Every organization reviewed, ASAM, SAHM, AAP, AACAP, BCCSU, and the AHRQ/Brown EPC, endorses buprenorphine for adolescents with OUD [1-4,6,7]. Although their emphases differ, the convergence of recommendations is striking: adolescents should receive MOUD without delay, without psychosocial prerequisites, and without age‑based restrictions.

Multiple organizations explicitly state that psychosocial treatment, while valuable, must function as a gatekeeper to medication. ASAM notes that the absence of psychosocial services or a youth’s decision to decline them should not delay access to buprenorphine [1]. SAHM is even more direct, stating that adolescents and young adults who do not pursue behavioral therapy “should not be denied MOUD” [6].

Evidence for Safety, Effectiveness, and Longer‑Duration Treatment

Across major professional societies, buprenorphine is consistently identified as a safe and effective treatment for adolescent OUD. ASAM notes no evidence of major safety concerns in younger patients [1], and AAP highlights randomized trials demonstrating improved abstinence and treatment engagement [2]. AACAP emphasizes that medications for OUD are the only interventions with demonstrated mortality benefit for youth [3]. The AHRQ/Brown EPC review provides the strongest federal evidence base: longer courses of buprenorphine (2-3 months) increased abstinence more than fourfold compared with short tapers, improved retention, reduced opioid use, and decreased high‑risk behaviors, with no serious adverse events [7]. Reflecting these findings, several organizations, including AACAP and the AHRQ/Brown EPC review, explicitly recommend longer‑duration buprenorphine treatment and caution against short tapers, underscoring that sustained therapy is superior across every meaningful clinical outcome.

Developmental and Ethical Considerations

Guidelines acknowledge the developmental, legal, and ethical complexities of treating minors, including confidentiality, parental involvement, and state‑specific consent laws. However, they uniformly stress that these complexities must not limit access to effective care. ASAM and AAP emphasize developmentally appropriate, trauma‑informed, and youth‑centered approaches, while BCCSU highlights the importance of continuity of care as adolescents transition into young adulthood [1,2,4].

Extended‑Release Buprenorphine: Emerging Guidance and Persistent Misconceptions

Although evidence remains limited, BCCSU’s 2024 bulletin acknowledges that extended‑release buprenorphine may be appropriate for individuals aged ≤18 years when supported by clinical judgment. This represents an important policy signal, particularly for adolescents at high risk of nonadherence, diversion, or treatment dropout [5].

At the same time, several professional guidelines continue to reference outdated regulatory language that implies only transmucosal buprenorphine is “approved” for adolescents, inadvertently reinforcing the perception that extended‑release formulations are inappropriate or off‑limits for youth. These residual artifacts of earlier labeling language do not reflect current prescribing information and may contribute to clinician hesitancy in considering extended‑release options, even when clinically indicated.

Taken together, contemporary guidelines convey a consistent message: evidence‑based pharmacotherapy should be readily available to adolescents with OUD, and age alone should not limit access. This consensus provides a foundation for expanding treatment options, including the thoughtful use of extended‑release buprenorphine.

Rationale for extended‑release buprenorphine

Why Formulation Matters for Adolescents With OUD

Despite substantial evidence that MOUD reduce overdose, infectious disease transmission, and a wide range of social, educational, and legal harms, opioid agonist therapy in adolescents remains constrained by stigma, hesitation, and structural barriers. Families often struggle with the idea of “treating opioids with opioids,” and many states impose additional consent requirements for minors, reflecting persistent discomfort with pharmacotherapy. Yet the medical reality is unequivocal: untreated OUD in adolescents carries serious and immediate risks, and MOUD, particularly buprenorphine, remains the most effective intervention available.

Emerging evidence and clinical experience suggest that formulation matters, particularly for adolescents. Transmucosal buprenorphine is effective, but its success depends on daily self‑administration, an expectation that collides with the developmental and social realities of adolescence. Many young people with OUD lack stable routines, private spaces, or consistent supervision. Daily dosing requires executive‑functioning skills that are still developing, and transmucosal films or tablets can be lost, forgotten, or diverted. For some adolescents, diversion is a survival strategy; for others, it is an inadvertent risk to opioid‑naïve peers. These vulnerabilities are among the most common reasons families hesitate to initiate MOUD for their children.

Evidence Highlighting the Limits of Daily Transmucosal Buprenorphine

The recent study by Kleinman et al. in JAMA Pediatrics [8] illustrates why concerns about daily transmucosal dosing are clinically significant. Buprenorphine‑naloxone was associated with a 16% lower overdose hazard than methadone, and no deaths occurred among youth receiving buprenorphine. Yet despite these safety advantages, retention on transmucosal buprenorphine was shorter: the median time to discontinuation was only 30 days, compared with 67 days for methadone. Kleinman and colleagues call for the evaluation of extended‑release buprenorphine as a strategy to improve retention in youth. Their findings reinforce an important finding: buprenorphine is effective when adolescents remain on treatment, and the challenge is sustaining engagement long enough for them to benefit.

Developmental Alignment of Extended‑Release Buprenorphine

Extended‑release buprenorphine is well‑suited to address vulnerabilities that undermine adolescent adherence. Unlike daily transmucosal formulations, depot injections eliminate the need for daily decision‑making, remove the possibility of diversion, and ensure continuous therapeutic coverage during periods when adolescents are most unstable and most at risk. These advantages are developmentally aligned. Adolescents are more impulsive, more susceptible to peer influence, and more likely to experience family conflict, school disruption, or brief incarceration. Extended‑release buprenorphine bypasses these challenges by embedding adherence into the delivery system [12,47-58].

Differences Between Available Extended‑Release Formulations

Extended‑release buprenorphine is not a single product; the U.S. market includes two distinct dosage‑form architectures: (i) Sublocade from Indivior Inc (NDA #209819): monthly injections only, available in 100 mg and 300 mg strengths [29]; (ii) Brixadi from Braeburn Inc (NDA #210136): weekly dosing (8 mg, 16 mg, 24 mg, 32 mg) and monthly dosing (64 mg, 96 mg, 128 mg), with an additional 160 mg monthly strength authorized in the EU, UK, Australia, and several Middle Eastern and North African countries [30].

While no head‑to‑head comparisons exist, these formulations differ in clinically meaningful ways. For adolescents suboptimally controlled on the 100 mg monthly maintenance dose of Sublocade, the only escalation is to 300 mg monthly. Device characteristics also differ: the Indivior product uses a needle with a diameter approximately 67% larger and a length 25% longer than the weekly-monthly product from Braeburn [29,30].

**Figure 1:**
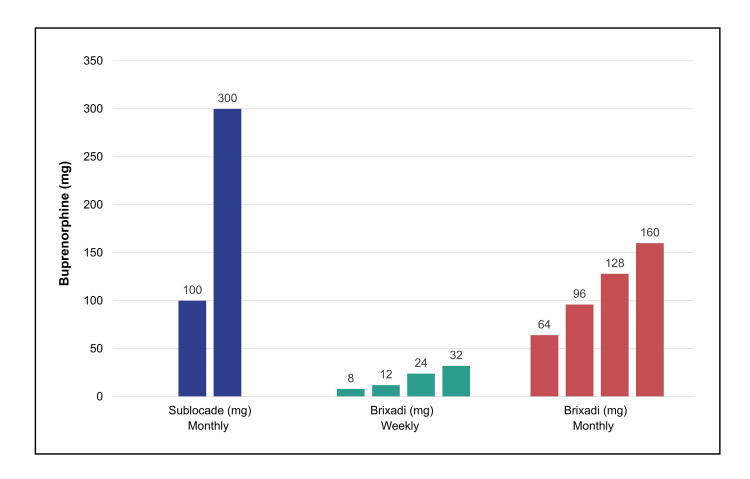
Extended‑Release Buprenorphine Dosage Forms from Indivior (Sublocade) and Braeburn (Brixadi) Extended‑release buprenorphine dosage forms: monthly Sublocade (Indivior Inc., NDA #209819) and weekly and monthly Brixadi (Braeburn, NDA #210136). Bar colors correspond to product groups: Sublocade in deep indigo, weekly Brixadi in teal blue, and monthly Brixadi in soft brick‑red. The 160‑mg monthly strength of Brixadi (marketed as Buvidal outside the U.S.) is not currently available in the U.S. but is authorized in the EU, UK, Australia, and a number of North African and Middle Eastern countries. Data derived from the U.S. Prescribing Information for Sublocade  [29] and Brixadi [30].

A formulation available for both weekly and monthly dosing in a wide range of strengths offers substantially greater potential for titration flexibility, allowing clinicians to adjust dose intensity more gradually, an important consideration for adolescents who may require stepwise stabilization. These design features matter because they enable clinicians to tailor treatment to developmental needs and clinical response rather than forcing large, abrupt dose escalations (Figure 1).

On April 30, 2026, Indivior announced it would discontinue Phase 3 development of INDV‑6001, a sustained‑release buprenorphine prodrug, citing internal R&D reprioritization. This decision suggests that no additional extended‑release buprenorphine formulations are likely to enter late‑stage development in the near term, further underscoring the importance of optimizing the clinical use of the two currently available depot buprenorphine products for adolescents.

Real‑World Outcomes Supporting Extended‑Release Buprenorphine

In a 2025 mirror‑image study across four Swedish opioid agonist treatment clinics, Gauffin et al. [48] followed healthcare utilization in 128 patients one year before and after switching from daily transmucosal buprenorphine to weekly or monthly extended‑release formulations. The switch cut inpatient days by half (9.0 to 4.5 days per year) and produced a 53% decrease in healthcare costs, with even greater reductions among patients retained on therapy. One‑year retention was 67%. These findings counter concerns that injectable formulations increase instability and instead show that extended‑release buprenorphine improves continuity, reduces crises, and supports more stable recovery trajectories. Although conducted in adults, these results are directly relevant to adolescents, who face even higher risks of dropout, relapse, and treatment disruption.

Emergency Department-Initiated Extended‑Release Buprenorphine: Evidence From the Fentanyl Era

Additional support for extended‑release formulations comes from the recent ED‑INNOVATION randomized clinical trial by D’Onofrio et al. [59], the largest head‑to‑head comparison of extended‑release and transmucosal buprenorphine conducted to date. In this multicenter study of 1,994 adults presenting to 29 U.S. emergency departments, most with fentanyl exposure, polysubstance use, unstable housing, and other markers of clinical complexity, seven‑day extended‑release buprenorphine was as effective as transmucosal buprenorphine for short‑term treatment engagement at 7 and 30 days. Although the primary endpoint was null, extended‑release buprenorphine demonstrated several clinically meaningful advantages: lower cravings, fewer days of illicit opioid use, higher treatment satisfaction, and better patient‑reported functioning across multiple domains. Importantly, precipitated withdrawal was rare (<1%) in nearly 2,000 buprenorphine initiations despite widespread fentanyl exposure, directly countering concerns that fentanyl precludes safe induction. These findings highlight that extended‑release buprenorphine is feasible, well‑tolerated, and clinically valuable in high‑risk, hard‑to‑reach populations, features directly relevant to adolescents, who face similar instability, high overdose risk, and substantial barriers to daily medication adherence.

Adherence as the Mechanism of Benefit

Across multiple studies, adherence to buprenorphine is one of the strongest predictors of retention, and retention is the mechanism through which MOUD exerts its effects. Viera et al. [60] concluded that adherence is positively associated with retention and that younger age consistently predicts shorter treatment duration. Matson et al. [61] found that retention correlated with urine drug screens negative for opioids and positive for buprenorphine; youth who actually took their medication were the ones who stayed in care. Warden et al. [62] showed that early adherence predicted long‑term retention, while early opioid‑positive urine tests sharply increased dropout risk. These findings converge on a single principle: adherence is not optional; it is the hinge on which treatment success turns. Extended‑release buprenorphine appears uniquely designed to protect this hinge. 

Clinical and Family‑Centered Advantages

In adults, extended‑release formulations maintain stable therapeutic levels, reduce illicit opioid use, and virtually eliminate diversion because no take‑home supply exists. For adolescents whose daily lives may be unpredictable or disrupted by housing instability or justice involvement, these features are especially protective. A clinician‑administered weekly or monthly injection provides continuous receptor coverage during the most unstable moments of early recovery, when overdose risk is highest.

For families, extended-release buprenorphine reframes treatment. Instead of sending a minor home with a controlled substance, clinicians administer a supervised medical intervention that prevents relapse and overdose. In states requiring parental consent, this distinction can be the difference between declining MOUD entirely and accepting a safer, more controlled option.

Patient Experience and Developmental Fit

Ariano et al. [47] provide one of the most recent qualitative examinations of extended‑release buprenorphine, using a narrative‑medicine framework across Italian addiction services. Although conducted in adults, the findings highlight several advantages relevant to adolescents: improved autonomy, emotional stability, self‑esteem, daily functioning, reduced stigma, and strengthened therapeutic relationships. Participants reported fewer cravings, greater symptom control, and enhanced motivation for recovery. These experiential themes, reduced stigma, improved adherence, increased autonomy, and stabilization of daily life, mirror the developmental needs of adolescents.

Editorial perspectives echo this alignment. Calihan et al. [63] argue that extended‑release formulations may be uniquely positioned to overcome the adherence, stigma, and access barriers that undermine youth engagement. Monthly injections provide continuous treatment coverage without daily decision‑making, fulfill youth preferences for discreet and low‑burden medication formats, and may be particularly beneficial for adolescents facing unstable housing, chaotic routines, or limited caregiver support.

Federal Research Momentum: The ERA Trial

Federal research momentum is now catching up to the clinical rationale for extended‑release buprenorphine. The proposed NIH‑funded HEAL Initiative Extended‑release Buprenorphine for Adolescents (ERA) Trial (NIDA CTN 0158) represents an important milestone [24]. This 24‑week study will directly compare extended‑release buprenorphine with daily transmucosal buprenorphine in adolescents aged 14-21. The absence of a placebo or detoxification arm signals that clinical equipoise no longer exists around withholding MOUD from adolescents. However, it remains unclear which extended‑release product will be used, and the study population spans two regulatory age categories, adolescents (14-17) and young adults (18-21). Adolescents represent the population for whom an indication is most urgently needed. It is also unclear whether the trial will be adequately powered to permit meaningful age‑stratified analyses of adolescents and young adults.

Synthesis: A Mechanistic and Developmental Rationale

The absence of adolescent‑specific trials with extended‑release buprenorphine should be acknowledged, but it does not negate the strong mechanistic rationale supported by youth adherence‑retention data, adult extended‑release outcomes, the Kleinman findings [8], the developmental science of adolescence, and the lethality of the fentanyl era. The risks of untreated OUD in adolescents are immediate, severe, and too often fatal. Extended‑release buprenorphine offers a developmentally appropriate, adherence‑enhancing, diversion‑proof, and potentially lifesaving option for youth at the highest risk of treatment dropout.

In this context, the case for extended‑release buprenorphine in adolescents is not merely compelling; it is increasingly relevant.

Adherence, retention, and barriers to MOUD

Clinician and Clinic Resistance

Across two decades of research, a striking pattern has emerged: adolescents with OUD are the age group least likely to receive MOUD, least likely to remain on it, and most likely to be steered toward non‑pharmacologic treatment, even though they face the highest overdose risk and have the most to gain from early, sustained pharmacotherapy. The literature consistently identifies the same interconnected forces: clinician resistance, family and patient ambivalence, structural barriers, regulatory misconceptions, and developmental vulnerabilities that undermine adherence and retention.

Clinicians themselves remain one of the most significant barriers to adolescent MOUD access. In a statewide survey of addiction treatment staff in Georgia, Welsh et al. [19] found a steep, age‑graded decline in support for MOUD: while most staff endorsed buprenorphine for adults, support dropped for young adults and fell sharply for adolescents aged 16-17. Effect sizes were medium to large, reflecting a pervasive reluctance to offer medication to younger patients. Staff cited concerns about brain development, safety, and beliefs that adolescents should “try counseling first,” views that contradict every major medical society guideline. Notably, respondents who had received formal MOUD education were significantly more supportive of treating adolescents, underscoring that clinician hesitancy is not fixed but shaped by training, exposure, and the perceived legitimacy of adolescent MOUD. These findings suggest that clearer regulatory signals, such as an adolescent‑specific buprenorphine indication, may help normalize MOUD for youth and reduce clinician hesitancy.

**Figure 2:**
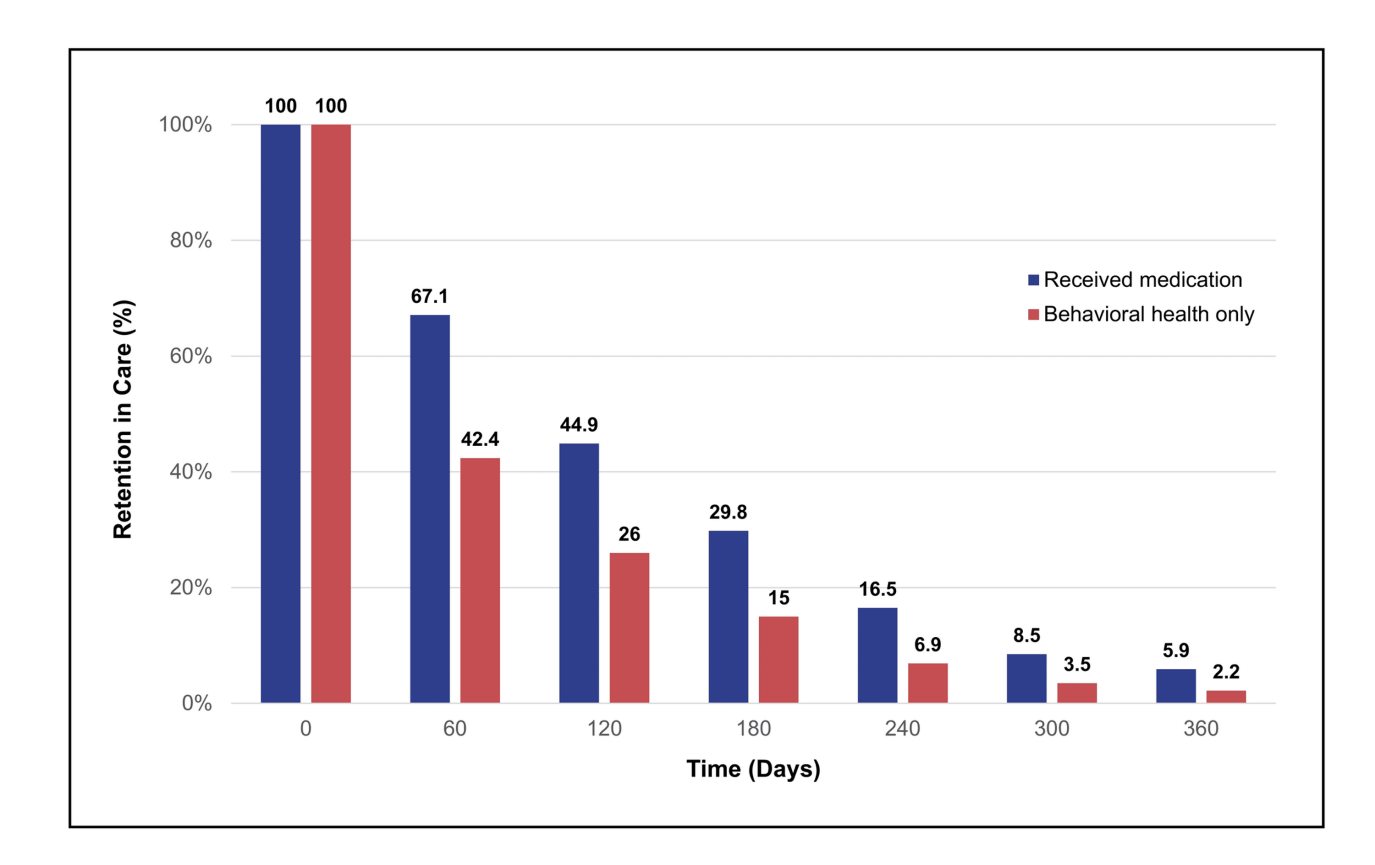
Retention in Care Among Medicaid‑Enrolled Adolescents and Young Adults With Opioid Use Disorder Retention in any addiction treatment at 0, 60, 120, 180, 240, 300, and 360 days among Medicaid‑enrolled adolescents (13–17 years) and young adults (18–22 years) with opioid use disorder, derived from the “No. at risk” data reported by Hadland et al. Deep indigo bars represent recipients of medication for opioid use disorder (MOUD), and soft brick‑red bars represent recipients of behavioral health services only. Data derived from the study by Hadland et al. [14].

This reluctance has measurable clinical consequences. Hadland et al. [14] found that adolescents and young adults who received MOUD soon after an OUD diagnosis remained engaged in treatment for substantially longer periods than those who received behavioral health services alone (Figure 2). Youths treated only with behavioral interventions had a median retention of 67 days, whereas those who initiated buprenorphine, naltrexone, or methadone remained in care for approximately 123, 150, and 324 days, respectively. A similar pattern was observed when examining the duration of behavioral health services specifically: individuals who did not receive timely MOUD had a shorter median duration (65 days) compared with those who received buprenorphine (108 days), naltrexone (152 days), or methadone (217 days). Together, these findings illustrate that clinician hesitancy does not simply limit access; it directly shortens treatment engagement.

Primary care settings reveal a similar pattern of structural and attitudinal barriers. Pippin [64] provides a granular look at these obstacles: among Louisiana family physicians, only 26% prescribed buprenorphine. The remaining 74% cited a constellation of concerns, fear of attracting “disruptive” patients, insufficient time to manage MOUD, lack of addiction‑specialist support, inadequate training, concerns about diversion, limited mental health resources, and lingering anxiety about DEA scrutiny. These findings echo national trends described by Schuler et al. [32], who show that buprenorphine prescribing has expanded for older adults but stagnated or declined for youth.

Even when system‑level reforms are implemented, they often fail to reach adolescents. Chavez et al. [65] showed that in the PROUD trial, nurse care management significantly increased MOUD access for adults but had no meaningful effect for youth aged 16-25. The intervention simply did not address the deeper developmental and structural barriers that uniquely affect adolescents.

Finally, many programs are not designed to serve adolescents at all. Yeh et al. [66] found that numerous treatment programs do not accept adolescents, and those that do often lack youth‑tailored services. Providers described feeling unprepared, unsupported, and unsure how to integrate MOUD into adolescent care. The result is a system where clinicians, intentionally or not, become gatekeepers who restrict access to the very medications that reduce overdose risk.

Patient and Family Ambivalence

Adolescents and their families also face internal barriers that shape treatment engagement. Buchholz et al. [67] found that while youth often describe buprenorphine as stabilizing and essential, many express ambivalence about long‑term medication use. They worry about being “on medication forever,” misunderstand the chronic nature of OUD, or fear stigma from peers and family. Caregivers, too, often begin with limited knowledge of MOUD and may initially prefer abstinence‑based approaches.

Yet the same study shows that once youth and caregivers understand the purpose of buprenorphine, they overwhelmingly view it as a lifeline. Extended‑release formulations were especially valued for reducing daily adherence burdens, an important insight given the developmental challenges adolescents face with routine medication taking.

These findings align with those of Viera et al. [60], who identified family conflict, parental substance use, and unstable home environments as major predictors of poor adherence and early dropout. Adolescents rarely have the autonomy, stability, or executive functioning to manage daily medication without strong support.

Profound Undertreatment

The result of these attitudinal and structural barriers is unmistakable: adolescents with OUD remain severely undertreated. Cano et al. [34] found that only 7.7% of adolescents with OUD received MOUD, compared with 19.3% of adults. Boggis et al. [33] reported that adolescents entering specialty treatment had just 5% of the odds of receiving MOUD compared with adults, one of the most extreme age‑based disparities documented in addiction medicine.

Gupta et al. [68] showed that in Indiana Medicaid, only 8.4% of diagnosed adolescents received MOUD, with even lower rates among Black youth. These findings mirror earlier work by Feder and others, demonstrating that adolescent MOUD access has remained stagnant for more than a decade. Hadland [14] similarly reported that most youths aged 13-22 enrolled in Medicaid received only behavioral health services (52.0%), with few receiving MOUD (23.5%); among adolescents specifically, only 4.7% received medication treatment. In commercially insured youth, Hadland et al. [13] found that 26.8% of individuals aged 13-25 were dispensed a medication within six months of OUD diagnosis. Younger adolescents were least likely to receive MOUD: among those aged 13-15, 16-17, 18-20, and 21-25 years, only 1.4%, 9.7%, 22.0%, and 30.5%, respectively, received MOUD.

Short Duration of Treatment

Even when adolescents do initiate MOUD, treatment duration is often strikingly short. Matson et al. [61] found that only 9% of youth remained in treatment at one year. Chavez et al. [65] reported median treatment durations of just 64-82 days. Viera et al. [60] showed that younger age consistently predicts shorter retention across studies.

**Figure 3:**
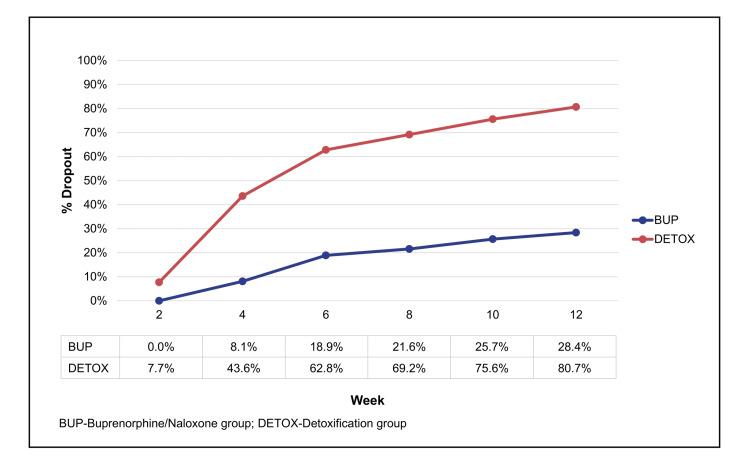
Timing of Attrition During 12-weeks of Buprenorphine and Detoxification in Adolescents and Young Adults Opioid-dependent adolescents and young adults (n=152), aged 15–21 randomized to 12 weeks of buprenorphine/naloxone (BUP, n=74) or two weeks of detoxification (DETOX, n=78) comprising a buprenorphine/naloxone taper, each in combination with 12 weeks of psychosocial treatment. In the DETOX group, 36% left between weeks 2 and 4, at the end of the dose taper, while in the BUP group, only 8% left by week 4. Reprinted from the study by Warden et al. with permission from Elsevier [62].

Warden et al. [62] demonstrated why this matters: early adherence predicts long‑term retention, and retention predicts abstinence. Youth who adhered to buprenorphine in the first two weeks were more likely to remain in treatment. Those who struggled early were at high risk of dropout, highlighting the need for early, intensive support (Figure 3).

Steering Adolescents Toward Non‑pharmacologic Treatment

A further pattern emerges: adolescents are routinely directed toward counseling‑only or abstinence‑based programs, even when they meet criteria for moderate to severe OUD. Welsh et al. [18] documented widespread staff beliefs that youth should “fail” behavioral treatment before receiving medication. Yeh et al. [66] found that many programs simply do not offer MOUD to adolescents at all.

Cano et al. [34] showed that adolescents are far more likely than adults to enter residential programs, settings historically less likely to offer MOUD. Boggis et al. [33] found that only 7.3% of adolescent treatment plans included MOUD, compared with 46% of adults.

This systematic steering away from medication contradicts the evidence. Hammond et al. [69] demonstrated that buprenorphine enhances, not replaces, therapy. Youth on buprenorphine attended more counseling sessions, formed stronger therapeutic alliances, and achieved higher abstinence rates. Medication enables engagement; it does not undermine it.

Regulatory Misconceptions and Policy Barriers

Regulatory misconceptions compound these clinical and structural barriers. To address the worsening overdose crisis, regulatory changes in 2023 expanded access to naloxone and buprenorphine, including over‑the‑counter naloxone and removal of the waiver requirement to prescribe buprenorphine for OUD. Early indicators are encouraging; adolescent overdose deaths declined by approximately 50%, yet mortality in 2023 remained more than twice as high as pre‑pandemic levels [70-73]. Despite these policy advances, treatment engagement remains strikingly low: fewer than one‑third of youths receive timely addiction treatment after an opioid overdose, and only 1 in 54 receive recommended pharmacotherapy [74].

Unfortunately, no buprenorphine formulation is FDA‑approved for OUD in individuals under 18. This lack of indication fuels provider hesitancy, reinforces stigma, and creates administrative barriers, shaping clinician behavior, insurer policies, and program eligibility. It also contributes to racial and age‑based disparities, as shown in the study by Gupta et al. [68], where younger adolescents and Black youth were far less likely to receive MOUD.

Despite this, the evidence is clear: buprenorphine is safe, effective, and developmentally appropriate for adolescents. Every major medical organization, including AAP, ASAM, SAHM, WHO, and BCCSU, endorses its use in youth. The barrier is not evidence; it is a slow regulatory adaptation.

A System Designed for Adults Cannot Meet the Needs of Adolescents

Taken together, these findings reveal a treatment system fundamentally misaligned with the developmental realities and clinical needs of adolescents. At every stage, from diagnosis to treatment initiation to long‑term engagement, youth encounter barriers that are qualitatively different from those faced by adults, yet the system continues to operate as if adolescents can simply be folded into adult models of care.

Clinician hesitancy, family ambivalence, structural limitations, and regulatory misconceptions converge to create a system in which adolescents are the least likely to receive the only intervention proven to reduce overdose mortality. The result is a paradox: adolescents face increasing overdose risk yet are the only group systematically excluded from the medications that may reduce overdose-related mortality.

Morbidity and mortality in adolescent OUD

The Opioid Overdose Crisis

**Figure 4:**
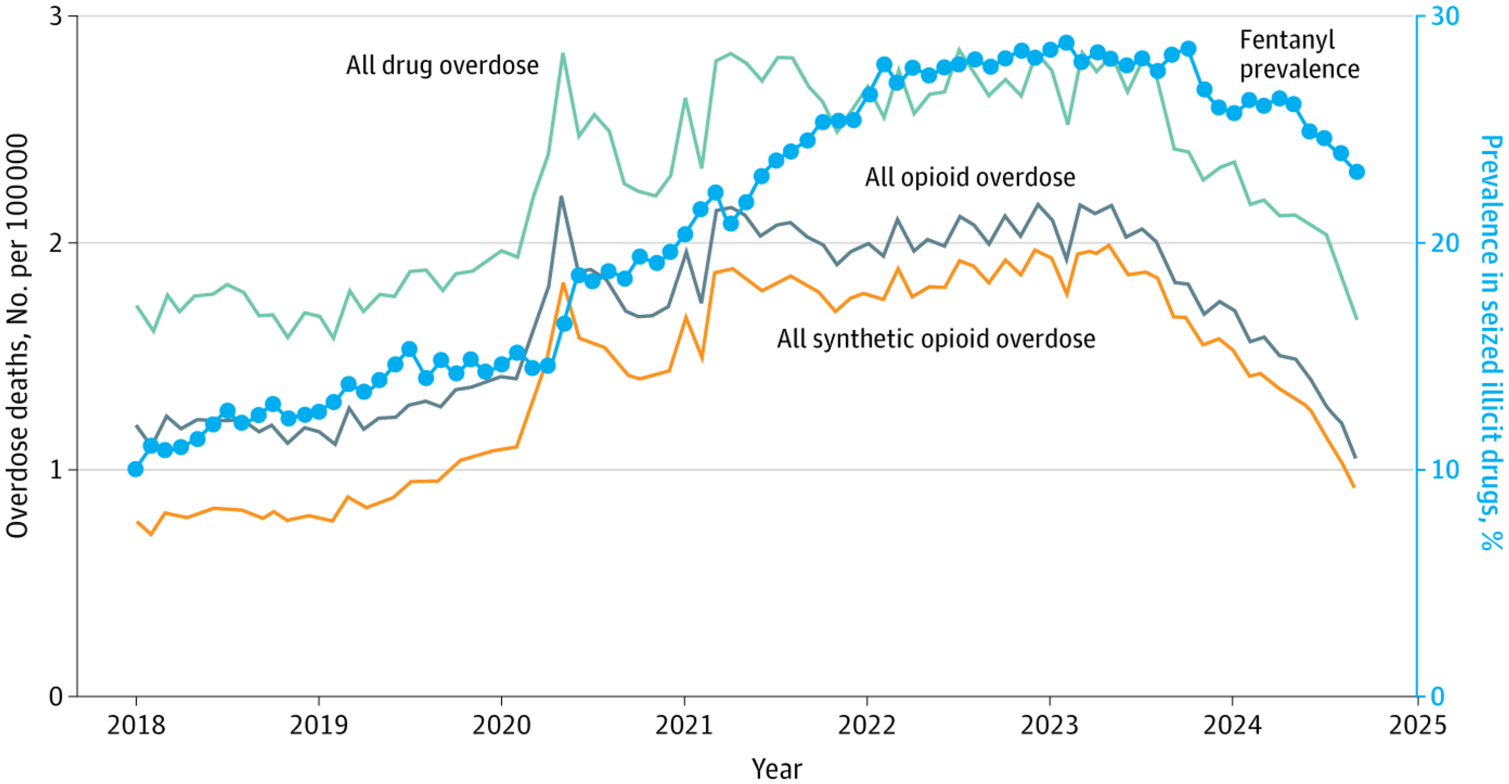
National Trends in Monthly Overdose Deaths per 100,000 and the Proportion of Fentanyl in the Illicit Drug Supply Trends in overdose deaths and the proportion of fentanyl reports in illicit drug seizures nationally. Opioid overdose mortality tracked closely with the proportion of fentanyl detected in law‑enforcement drug seizures. Both indicators increased through the winter of 2022 and subsequently began to decline. Reprinted from the study by Dahlen et al. under the terms of the CC‑BY license [72].

After a substantial increase in adolescent overdose deaths, driven almost entirely by the lethality of illicitly manufactured fentanyl (IMF), recent years have shown a partial reversal, with age‑adjusted overdose death rates declining between 2022 and 2024, and the largest decreases occurring from 2023 to 2024, particularly among younger age groups. National mortality data for 2025 have not yet been released, so current trends can only be assessed through 2024. Between 2023 and 2024, the drug overdose death rate from synthetic opioids decreased by 30% to 50% (Figure 4). Nearly 90% of all adolescent overdose deaths involve IMF. Counterfeit pills, often indistinguishable from legitimate medications, were implicated in one‑quarter of deaths, highlighting that many adolescents who die from overdose are opioid‑naïve or unaware they are consuming fentanyl [70,73-79].

These deaths frequently occur in the presence of potential bystanders; two‑thirds of decedents had someone nearby, but most received no response, often because the overdose was silent, unexpected, or unrecognized [78]. This pattern underscores a critical reality: adolescents are not dying from chronic heroin addiction but from sudden, unanticipated fentanyl exposure, often during experimentation or early misuse. The window for intervention is narrow, and the consequences of delayed treatment are severe.

Recent pediatric emergency department data reinforce this pattern. Devlin et al. [80] analyzed 4,550 overdose visits across 42 children’s hospitals between 2017 and 2022 and found rising severity, a sharp increase in ICU admissions, and widening racial inequities, with adolescent overdoses increasingly involving fentanyl. The authors highlight a critical disconnect: pediatric overdose deaths quadrupled since 2018, yet ED visits rose far less, reflecting the rapid lethality of fentanyl and the need for community‑level naloxone distribution and youth‑centered treatment pathways.

A Treatment System Misaligned With Adolescent Overdose Risk

Despite this escalating risk, adolescents remain the age group least likely to receive evidence‑based treatment for OUD. Schuler et al. [32] show that while buprenorphine access expanded for adults between 2007 and 2018, adolescents experienced an absolute decline in treatment episodes. Bagley et al. [81] found that only 14% of adolescents diagnosed with OUD in primary care received any medication, compared with 32-39% of young adults. Cano et al. [34] and Boggis et al. [33] report similarly stark findings: across national datasets, only 5-10% of adolescents with OUD receive MOUD, compared with 30-50% of adults.

These disparities persist even though adolescents face a substantial overdose risk and the least predictable patterns of opioid exposure. The treatment system designed around adult models of chronic opioid dependence has not adapted to the fentanyl era, in which adolescents require rapid stabilization, continuous receptor coverage, and developmentally appropriate care.

Clinical Consequences of Undertreatment

The consequences of this treatment gap are significant. Mintz et al. [35] demonstrated that adolescents have the lowest retention of any age group, with only 17.6% remaining in care at six months. Yet this age‑related disparity nearly disappears when adolescents receive buprenorphine. Adolescents treated with buprenorphine were more than four times as likely to remain in treatment compared with those receiving psychosocial care alone. Medication, not age, is the decisive factor in whether adolescents remain engaged long enough to benefit from treatment.

Warden et al. [62] further showed that early adherence predicts long‑term retention, and retention predicts abstinence. Adolescents who adhered to buprenorphine in the first two weeks were significantly more likely to remain in treatment; those who struggled early were at high risk of dropout. These findings highlight the need for early, intensive support and for treatment models that minimize daily adherence demands.

Why Buprenorphine Is Essential in the Fentanyl Era

Buprenorphine’s pharmacologic properties make it protective for adolescents exposed to fentanyl. As emphasized by Ball et al. [31], buprenorphine’s high receptor affinity and partial agonism blunt the effects of fentanyl and reduce overdose risk. When buprenorphine occupies the mu‑opioid receptor, it prevents fentanyl from binding with full‑agonist potency, creating a pharmacologic “buffer” that reduces lethality. In adults, MOUD reduces mortality by approximately 50%; for adolescents navigating a drug supply dominated by counterfeit fentanyl‑containing pills, the protective effect may be even more critical.

Chang et al. [82] argue that buprenorphine should be considered first‑line treatment for adolescents with OUD, emphasizing that psychosocial treatment alone is insufficient and that detoxification‑only approaches expose youth to relapse and overdose. They cite randomized trials by Marsch et al. [10,11] and Woody et al. [9] showing consistently improved retention, reduced cravings, and lower relapse rates compared with non‑agonist strategies. They emphasize that youth require longer stabilization periods, not short tapers, and recommend treatment durations exceeding 52 weeks. Their guidance is unequivocal: “When in doubt, do not taper.”

Structural and Regulatory Barriers That Exclude Adolescents

Yet despite strong evidence and professional consensus, adolescents remain systematically excluded from MOUD. Ruth et al. [83] found that clinicians frequently misunderstand consent laws, overestimate regulatory barriers, and hold stigmatizing beliefs about adolescents with OUD. Although no buprenorphine formulation is FDA‑approved for the treatment of OUD in individuals under 18, substantial evidence supports its use in this population, and major organizations, including ASAM [1], AAP [2], AACAP [3], NIDA [39], AHRQ [7], and SAHM [6,40], explicitly recommend buprenorphine for adolescents with OUD. Many clinicians remain unaware of this consensus or feel unprepared to manage adolescent OUD, even after elimination of the federal waiver requirement.

These misconceptions are compounded by the lack of an FDA indication for buprenorphine in individuals under 18. Although this absence reflects historical research gaps, not evidence of harm, it fuels clinician hesitancy, insurer restrictions, and programmatic exclusion. The result is a paradox: adolescents face an increasing overdose risk yet are the only group who experience markedly reduced access to the medications that may reduce overdose‑related mortality.

Federal Efforts to Close the Evidence Gap

Recognizing the urgent need for adolescent‑specific data, the NIH HEAL Initiative is funding the Extended‑Release Buprenorphine for Adolescents (ERA) Trial (CTN‑0158), the first large, multisite study designed to compare the relative effectiveness and adherence of extended‑release buprenorphine with standard transmucosal formulations in adolescents and young adults aged 16-21 [24]. Although the trial has not yet begun recruitment, its development signals a major federal commitment to addressing the adolescent MOUD evidence gap.

A Public Health Imperative

The cumulative evidence paints a clear and increasingly relevant picture. Adolescents are dying at unprecedented rates from fentanyl exposure. They are the least likely age group to receive MOUD, the least likely to remain in treatment without it, and the most vulnerable to the consequences of inadequate care. The scientific literature is clear: buprenorphine is safe, effective, and lifesaving for adolescents with OUD. The barriers to its use, regulatory misconceptions, clinician hesitancy, structural limitations, and outdated treatment paradigms are not grounded in evidence but in inertia.

In the fentanyl era, failing to provide adolescents with timely access to buprenorphine is not a neutral clinical decision; it is a missed opportunity to prevent avoidable deaths. The epidemiology demands a recalibration of policy, practice, and regulatory frameworks to ensure that adolescents receive the same evidence‑based treatment routinely offered to adults.

Evidence from case reports, clinical trials, and reviews

Overview

A remarkably consistent evidence base has accumulated over almost 20 years, spanning randomized trials, observational cohorts, administrative datasets, qualitative studies, and narrative reviews: buprenorphine is safe, feasible, and effective for adolescents and young adults with OUD. The earliest randomized trials [9,10] established the foundational principles that continue to guide adolescent OUD treatment today: buprenorphine improves retention, reduces opioid use, decreases high‑risk behaviors, and is well tolerated in youth. These trials also demonstrated that short tapers are clinically inferior, with rapid relapse and early dropout, and that longer treatment durations yield better outcomes [11].

**Figure 5:**
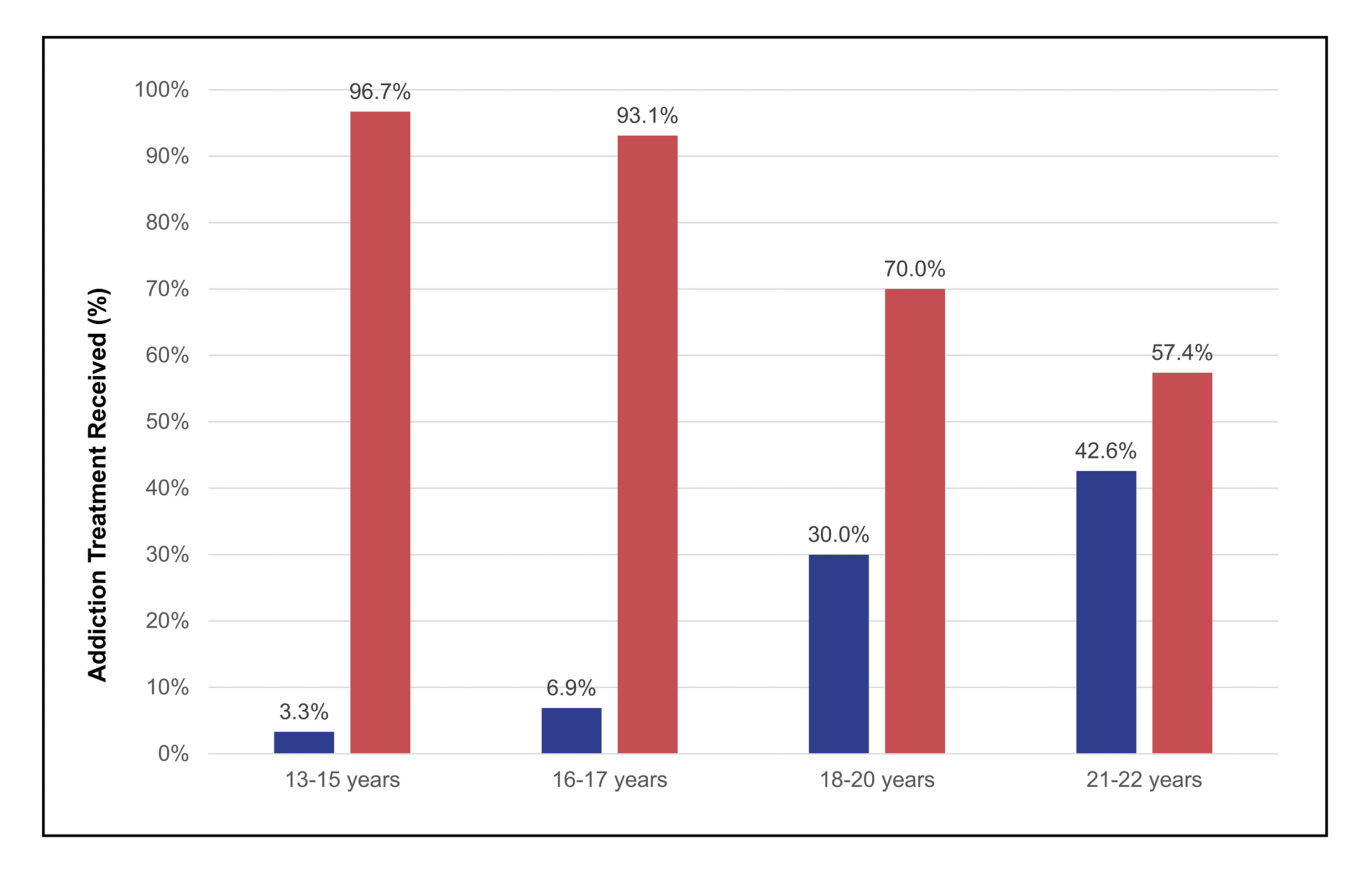
Receipt of Addiction Treatment Within Three Months of OUD Diagnosis by Age Group Medicaid‑enrolled youth with OUD (n = 4,837), categorized into age groups of 13-15, 16–17, 18–20, and 21–22 years. Deep indigo bars represent receipt of MOUD, with or without concurrent behavioral health services (n = 1,139). Soft brick‑red bars represent receipt of behavioral health services only (n = 2,515). MOUD included buprenorphine, naltrexone, or methadone. Data derived from a table in the study by Hadland et al. [14]. OUD: Opioid use disorder; MOUD: Medications for OUD

As fentanyl reshaped the risk environment, real‑world studies expanded the evidence base. Large administrative datasets [13,14,33,34,68] consistently show that adolescents are the age group least likely to receive MOUD, despite facing escalating overdose risk (Figure 5). Yet when youth do receive buprenorphine, retention improves, relapse decreases, and engagement stabilizes. Comparative effectiveness research [8] further demonstrates that buprenorphine is at least as effective, and likely safer than methadone for youth.

Contemporary fentanyl‑era studies [12,63,84] demonstrate that adolescents can successfully initiate buprenorphine even when using illicit fentanyl exclusively, directly countering the misconception that fentanyl precludes safe or effective induction. Narrative reviews and scoping analyses [31,85-87] integrate these findings into a unified conclusion: buprenorphine is the only MOUD with randomized trial evidence in adolescents, and withholding it is harmful. System‑level studies [19,32,64,66] show that undertreatment stems from structural barriers, clinician hesitancy, lack of pediatric prescribers, regulatory misconceptions, stigma, and programmatic exclusion of minors, not from lack of efficacy.

Adult ED‑based randomized trials, including the recent ED‑INNOVATION study by D’Onofrio et al. [59], further demonstrate that extended‑release buprenorphine is safe, feasible, and well‑tolerated even in fentanyl‑dominant populations, reinforcing its potential relevance for adolescents.

Additional real‑world evidence comes from Kaliamurthy et al. [88], who described 24 adolescents with fentanyl‑positive toxicology at intake and found that 88% initiated or continued MOUD, with encouraging early retention. The study highlights that adolescents and families overwhelmingly accept buprenorphine when offered, and that lack of psychosocial engagement should not delay medication initiation in the fentanyl era.

Taken together, the evidence demonstrates that expanding access to both transmucosal and extended‑release buprenorphine is essential to reducing overdose deaths in youth. Adolescents remain undertreated despite a strong evidence base, and failure to provide MOUD contributes directly to preventable harm.

Randomized Controlled Trials

Randomized trial evidence for adolescent buprenorphine treatment, though limited in number, is remarkably consistent. Marsch et al. [10] conducted the first landmark RCT demonstrating that buprenorphine was substantially superior to clonidine for adolescents with opioid dependence, producing higher retention (72% vs 39%), more opioid‑negative urines, and better withdrawal control. Building on this foundation, Woody et al. [9] reported that in the largest adolescent/young adult RCT to date (N=152), a 12‑week buprenorphine-naloxone maintenance regimen outperformed a 14‑day taper, with retention of 70% versus 20.5% and substantially reduced opioid use, injecting, and polysubstance use.

Subsequent work reinforced this dose‑duration effect. Gonzalez et al. [89] found that memantine augmentation improved abstinence after buprenorphine discontinuation among young adults, underscoring the vulnerability associated with early tapering. Marsch et al. [11] extended these findings to minors aged 16-17, demonstrating that a 56‑day taper produced significantly more opioid‑negative urines than a 28‑day taper. Becker et al. [87] synthesized all available RCTs and concluded that buprenorphine is the only MOUD with randomized‑trial evidence in adolescents and that longer treatment durations consistently yield better outcomes.

Observational, Cohort, and Administrative Studies

Real‑world data paint a stark picture of undertreatment. Hadland et al. [13] showed that only 1.4% of 13-15‑year‑olds and 9.7% of 16-17‑year‑olds with OUD received MOUD, despite buprenorphine accounting for nearly 90% of prescriptions. Among 2.4 million adolescents (13 to 17) and young adults (18 to 22) from 11 states enrolled in Medicaid [14], timely buprenorphine initiation nearly doubled retention, yet only 4.7% of adolescents received MOUD, compared to 26.9% of young adults. Adolescents were more likely to receive naltrexone than were young adults (35.3% vs. 11.1%). Schuler et al. [32] reported that adolescents represented just 0.1% of 4.1 million buprenorphine treatment episodes, even as youth overdose deaths rose sharply. More recently, Hadland et al. [17] analyzed 11,649 Massachusetts youth initiating buprenorphine. Only 24% achieved high adherence for 12 months, and this group had the lowest subsequent risk of overdose, emergency department use, and hospitalization, reinforcing that sustained buprenorphine treatment is protective even in real‑world settings.

Qualitative and case‑based evidence reinforces these patterns. Schmuhl et al. [90] documented successful extended‑release buprenorphine treatment in a 17‑year‑old, while Buchholz et al. [67] found that youth and caregivers overwhelmingly view buprenorphine as stabilizing and developmentally appropriate, with a strong preference for extended‑release formulations. Administrative datasets deepen the picture: Cano et al. [34] reported that only 7.7% of adolescents with OUD receive MOUD nationally; Boggis et al. [33] found that adolescents in specialty treatment had only 5% of the adjusted odds of receiving MOUD compared with adults; and Gupta et al. [68] identified profound racial inequities, with Black adolescents 89% less likely than White peers to receive medication. Taken together, these studies underscore the need for interventions that enhance medication adherence among adolescents with OUD, particularly given persistently low rates of MOUD initiation [13,14], including close clinical follow‑up when medication is discontinued, naloxone dispensing, family counseling about the high risk of relapse and overdose with early discontinuation, and consideration of extended‑release buprenorphine for youth who struggle with daily adherence.

Comparative Effectiveness

Strong population‑level evidence comes from Kleinman et al. [8], who compared buprenorphine-naloxone with methadone among 6,842 youths aged 15-24. Buprenorphine was associated with lower overdose risk (HR 0.84; per‑protocol HR 0.42), and eight deaths occurred during methadone treatment versus none on buprenorphine. Despite shorter retention on buprenorphine, its safety profile strongly favors it as first‑line therapy for youth.

Complementing these findings, Jerry et al. [49] demonstrated that sustained adherence to extended‑release buprenorphine is associated with markedly reduced healthcare utilization and non‑MOUD medical costs. Patients adherent to extended‑release buprenorphine had substantially fewer inpatient admissions, ED visits, and detoxification episodes, underscoring the stabilizing effect of long‑acting formulations and their potential value for adolescents who struggle with daily adherence.

Fentanyl‑Era Feasibility and Induction Studies

Contemporary studies demonstrate that fentanyl exposure does not preclude successful buprenorphine initiation. Wenzel & Fishman [91] demonstrated that mobile extended‑release buprenorphine delivery improves engagement among youth. Trope et al. [84] showed that 87% of fentanyl‑using adolescents successfully initiated buprenorphine in an inpatient program, with 72% linking to ongoing MOUD. Neptune & Kaliamurthy [12] reported successful extended‑release buprenorphine treatment in six adolescents aged 15-17. Calihan et al. [63] emphasized that extended‑release formulations align with adolescent developmental needs and reduce stigma.

System‑Level Barriers and Workforce Limitations

Structural barriers, not lack of efficacy, explain why adolescents remain undertreated. Yeh et al. [66] found that adolescent OUD programs are nearly nonexistent and that adult‑oriented models are developmentally misaligned. Pippin et al. [64] identified clinician hesitancy, lack of training, and regulatory anxiety as major barriers. McCarty et al. [86] highlighted insurance restrictions and behavioral‑therapy prerequisites. Hadland et al. [16] reported that only 12.4% of pediatricians feel responsible for prescribing MOUD. Chavez et al. [65] showed that adult‑focused system interventions fail to improve youth MOUD access.

Prevention infrastructure faces similar constraints. Kuklinski et al. [92] quantified the substantial startup costs required to implement opioid‑misuse prevention programs across diverse youth‑serving systems, highlighting that training, partner engagement, and workflow development account for the majority of expenditures. These findings underscore that without dedicated funding, even evidence‑based prevention models struggle to achieve sustainable implementation.

Narrative Reviews and Conceptual Papers

Narrative syntheses reinforce the empirical evidence. Welsh et al. [21] and Ball et al. [31] provide comprehensive reviews of youth OUD epidemiology, emphasizing that despite modest recent declines in overdose deaths, adolescents remain undertreated and face escalating fentanyl‑driven risk. These reviews reaffirm that MOUD, particularly buprenorphine, markedly improve retention and reduce mortality in adolescents, mirroring outcomes seen in adults, yet real‑world uptake remains minimal due to stigma, limited youth‑focused programs, and inconsistent Medicaid acceptance. They also underscore that naloxone is highly effective in reducing overdose risk and unintentional death, with its impact strengthened by broader education and distribution. In a companion update, Welsh et al. [20] modernize national principles for adolescent SUD treatment, emphasizing developmental tailoring, family engagement, harm‑reduction integration, and the need for cross‑system coordination across schools, primary care, emergency departments, and community programs. They highlight that without structural and financial support, even the most evidence‑based practices fail to reach adolescents, the group at greatest long‑term risk. Although not youth‑specific, Beliveau & Baca‑Atlas [93] critique punitive urine drug screening practices that undermine engagement, issues that may be even more consequential for adolescents, who already face heightened barriers to MOUD access. Similarly, Fox et al. [94] describe low‑threshold buprenorphine models that reduce structural barriers and improve engagement, approaches that could help address the profound access challenges experienced by youth.

Modeling and Simulation Studies

Nataraj et al. [95] demonstrate that MOUD initiation is the single most powerful lever for preventing overdose deaths at the population level. Initiating 62.6% of untreated individuals annually could offset rising mortality. Adolescents, who have the lowest MOUD access, represent the largest untapped opportunity for mortality reduction.

## Conclusions

Adolescents with OUD remain substantially undertreated despite clear evidence that buprenorphine is safe, effective, and associated with markedly improved retention and reduced overdose risk. Across clinical trials, observational cohorts, and modeling analyses, longer and sustained treatment consistently outperforms short tapers or psychosocial approaches alone.

The findings of this review demonstrate that the primary barriers to adolescent access are structural and regulatory rather than evidence‑based. Aligning clinical practice and policy with the established scientific consensus, including broader availability of buprenorphine and consideration of extended‑release formulations, is essential to reducing preventable morbidity and mortality among youth with OUD.
